# Gender Differences in How Parents, Peers, and Exposure to Sexually Explicit Materials Influence the Intention to Engage in Casual Sex among Adolescents and Young Adults in Taiwan: Applying the Theory of Planned Behavior

**DOI:** 10.3390/ijerph182413089

**Published:** 2021-12-11

**Authors:** Ying-Hua Tseng, Wen-Li Hou, Shih-Hsien Kuo, Yu-Hsiang Liu, Hui-Ling Wang, Ray C. Hsiao, Fan-Hao Chou, Cheng-Fang Yen

**Affiliations:** 1 College of Nursing, Kaohsiung Medical University, Kaohsiung 80708, Taiwan; yhtseng@kmu.edu.tw (Y.-H.T.); wlhou422@gmail.com (W.-L.H.); 2 School of Nursing, Fooyin University, Kaohsiung 83102, Taiwan; ns215@fy.edu.tw (S.-H.K.); ns236@fy.edu.tw (Y.-H.L.); PT385@fy.edu.tw (H.-L.W.); 3 Department of Psychiatry and Behavioral Sciences, School of Medicine, University of Washington, Seattle, WA 98195, USA; rhsiao@u.washington.edu; 4 Department of Psychiatry, Children’s Hospital and Regional Medical Center, Seattle, WA 98105, USA; 5 Department of Psychiatry, School of Medicine College of Medicine, Kaohsiung Medical University, Kaohsiung 80708, Taiwan; 6 Department of Psychiatry, Kaohsiung Medical University Hospital, Kaohsiung 80756, Taiwan; 7 College of Professional Studies, National Pingtung University of Science and Technology, Pingtung 91201, Taiwan

**Keywords:** casual sex, theory of planned behavior, gender gap, parent, peer, sexually explicit material

## Abstract

The aims of this study were to examine gender differences in how parent–child discussions on sex issues, peer interactions around sexual issues, and exposure to sexually explicit materials affect the intention to engage in casual sex among adolescents and young adults in Taiwan. This cross-sectional survey study recruited 767 participants (348 men and 419 women) aged 15–24 years. The survey collected data on participants’ intention to engage in casual sex, their attitude toward and perception of casual sex based on the theory of planned behavior (TPB) (favorable attitude, perceiving positive social norms toward casual sex, and perceived control over involvement), parent–child and peer discussions about sexual issues, and exposure to sexually explicit materials. The results of multiple regression analysis revealed that parent–child discussions on sex issues, peer interactions around sexual issues, and exposure to sexually explicit materials were significantly associated with the intention to engage in casual sex. The results of structural equation modeling (SEM) further supported that favorable attitude, perceiving positive social norms toward casual sex, and control over involvement mediated the associations. For men, decreased favorable attitude mediated the negative association between parent–child discussions and casual sex intention; increased favorable attitudes and decreased control over involvement mediated the positive associations between peer interactions and casual sex intention. For women, decreased control over involvement mediated the positive association between exposure to sexually explicit materials and casual sex intention. The associations between peer interaction and subjective norms of acceptance, perceived control over involvement, and casual sex intention were stronger in men than in women; the association of favorable attitudes with casual sex intention was also stronger in men than in women.

## 1. Introduction

### 1.1. Casual Sex among Adolescents and Young Adults

Casual sex refers to sexual activity that occurs without expectations of a sustained and committed relationship [1]. Casual sex often results from sexual needs and physical attraction. Research has found that casual sex is prevalent among adolescents and young adults. The 2006–2007 Toledo Adolescent Relationships Study using the fourth wave dataset, originally collected in 2000 based on a random school sample of youth in Lucas County, Ohio, found that 59% of young adults (aged 18–24 years) had engaged in casual sex [2]. A study in Taiwan found that 14% of college students had experienced one-night stands [3]. Several studies have demonstrated the negative effects of casual sex among adolescents and young adults; risks include sexually transmitted diseases, physical and sexual aggression, negative emotional responses such as regret, depression, and anxiety, and conflicted interpersonal relationships [4,5,6,7,8]. However, young adults have reported positive effects of casual sex, including feeling sexual arousal and happiness and gaining confidence and closeness [8,9]. Further studies are needed to better understand the effects of, and factors related to casual sex so that intervention programs can be developed to improve sexual health in adolescents and young adults.

### 1.2. Theory of Planned Behavior

The theory of planned behavior (TPB) has been used to examine individuals’ sexual behaviors and intention to engage in safe sex [10,11]. According to the TPB, an individual’s behavior is mainly determined by behavioral intention; intentions, in turn, are determined by three constructs: attitude, subjective norms, and perceived behavioral control [10]. The construct of attitude refers to an individual’s specific beliefs about the anticipated costs and rewards of performing particular behaviors. The construct of subjective norms refers to an individual’s perception of social normative pressures on what behaviors should or should not be performed, and the construct of perceived behavioral control indicates an individual’s beliefs about the ease or difficulty of performing a behavior [10]. A previous study using the TPB revealed that a favorable attitude toward casual sex and perceiving a subjective norm accepting of casual sex were positively related to the intention to engage in casual sex, whereas perceived control over involvement negatively related to the intention to engage in casual sex [12]. Research has also found that past sexual behaviors, anticipated effects, perceived norms, and sexual inhibition influenced individuals’ attitude, subjective norms, and perceived behavioral control with respect to casual sex [12]. However, no studies have examined the influences of parents, peers, and exposure to sexually explicit materials on the intention to engage in casual sex and its TPB constructs.

### 1.3. Influences of Parents, Peers, and Exposure to Sexually Explicit Materials on Intention to Engage in Casual Sex

According to the ecological system theory [13], microsystemic factors such as parent–child discussions and peer interactions regarding sexual issues and exposure to sexually explicit materials may influence individuals’ intention to engage in casual sex. A well-functioning family plays a vital role in preventing risky sexual behaviors among adolescents [14]. A good parent–child relationship reduces the chance of a first sexual experience occurring in a casual relationship context among adolescents [15]. It is reasonable to hypothesize that sufficient parent–child discussions of sexual issues reduce the incidence of casual sex; however, the relationship between parent–child discussions of sexual issues (hereafter referred to as “parent–child discussion”) and its TPB constructs warrants further study.

Perceived peer sexual behavior norms have a major influence on decision-making regarding engaging in sex among adolescents and young adults in various cultures and countries [4]. One study found that adolescents and young adults who received more positive messages about casual sex from their peers had more sex partners, casual encounters, and sexual experiences [16]. Moreover, both dating apps [17,18] and pornographic media [19] increase the incidence of casual sex among adolescents and young adults. However, whether peer discussion concerning sexual issues (hereafter referred to as “peer interaction”) or exposure to sexually explicit materials and engagement with dating apps (hereafter collectively referred to as “exposure to sexually explicit materials”) influence the TPB constructs related to casual sex and the intention to engage in casual sex warrants further study.

### 1.4. Gender Differences

Research has shown that male adolescents and young adults have more casual sex experiences than women do [15,20,21]. There are also gender differences in the factors related to casual sex. For example, a healthy mother–son relationship was associated with a reduced risk of a male adolescent’s first sexual experience occurring in the context of a casual relationship, whereas a healthy father–daughter relationship and living in a two-parent family was associated with a reduced risk of a female adolescent’s first sexual experience being in a casual relationship [15]. Male young adults may strengthen peer connections by discussing sexual experiences, girlfriends, hook-ups, and one-night stands [22], whereas female young adults interact with peers to cope with gender inequality and increase their sexual autonomy [23]. However, no study has comprehensively examined gender differences in the roles of parent–child discussion, peer interaction, and exposure to sexually explicit materials in the intention to engage in casual sex and its TPB constructs. Studies on gender differences can provide evidentiary support for the development of gender-specific sexual health interventions.

### 1.5. Study Aims

**Figure 1:**
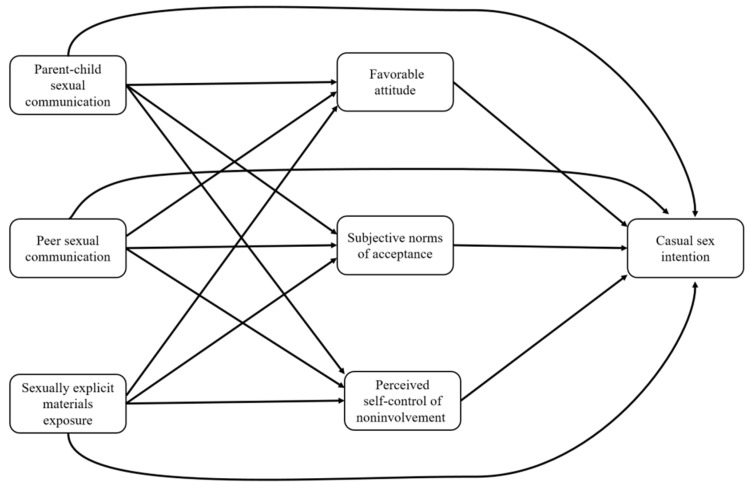
Proposed model in explaining intention to engage in casual sex.

This study had three aims. The first was to examine the influences of parent–child discussion on sex-related topics, peer interaction around sex-related topics, and exposure to sexually explicit materials on the intention to engage in casual sex and its TPB constructs, including a favorable attitude toward casual sex, perceiving positive social norms toward casual sex, and perceived control over involvement. We studied adolescents and young adults and used structural equation modeling (SEM). The second aim was to examine gender differences in how parent–child discussion, peer interaction, and exposure to sexually explicit materials influence the intention to engage in casual sex and its TPB constructs. Our hypotheses were as follows (Figure 1):

**Hypothesis** **1** **(H1).** *Parent–child discussion on sex-related topics is directly associated with reduced intention to engage in casual sex, whereas peer interaction around sex-related topics and exposure to sexually explicit materials are directly associated with increased intention to engage in casual sex.*

**Hypothesis** **2** **(H2).** *The three TPB constructs (favorable attitude toward casual sex, subjective norms of accepting casual sex, and perceived control over involvement) mediate the associations of parent–child discussion on sex-related topics, peer interaction around sex-related topics, and exposure to sexually explicit materials with the intention to engage in casual sex.*

**Hypothesis** **3** **(H3).** *Gender differences exist in the relationships among parent–child discussion on sex-related topics, peer interaction around sex-related topics, exposure to sexually explicit materials, the three TPB constructs, and the intention to engage in casual sex.*

## 2. Methods

### 2.1. Participants

This cross-sectional study was conducted in a college and two senior high schools of Taiwan in 2014. The researcher obtained permission from the school administrators to conduct the study. This study used a nonprobability sampling method to recruit participants. Five teachers in the three schools agreed to deliver the study information to the students in their classes. A total of 1185 students aged between 15 and 24 years were invited to participate in this study. The researcher explained the purpose of the study to the potential participants and to their parents or legal guardians using written instructions. Participants younger than 20 years old were required to obtain written parental consent to participate into the studies because they are legally considered as minors in Taiwan [24]. In total, 231 students who were 20 years of age or older agreed to participate, and 536 students who were younger than 20 agreed to participate with written parental consent. Participants were invited to complete the research questionnaires individually and anonymously. The questionnaire took 25 to 30 minutes to complete. The participants could ask questions clarifying questionnaire content, and research assistants answered them.

Due to the differences in intention and related behaviors between heterosexuals and non heterosexuals with regard to casual sex [25,26], the data of 62 participants who identified as homosexual or unsure were excluded. Because both father–child and mother–child discussions were assessed, the data of 17 participants whose father or mother had passed away were excluded. Moreover, the data of 339 participants who had missing data or answered carelessly on the questionnaires were excluded. Ultimately, the analysis included the data of 767 participants (348 men and 419 women). Simple structural equation models require a sample size of 200–500 [27]. This study was approved by the Institutional Review Board of Fooyin University (FYH-IRB-102-03-01). 

### 2.2. Measures

#### 2.2.1. Casual Sex and TPB Constructs Scale

This study used the Casual Sex and TPB Constructs Scale developed by Turchik and Gidycz [12] to assess attitudes, subjective norms, perceived behavioral control, and intention regarding casual sex. The questionnaire was translated from English to Chinese and back translation was used to verify the quality of the translation [28]. The scale contained three items measuring intention to engage in casual sex and 11 items measuring TPB constructs of casual sex. Casual sex was defined as “to have sexual behaviors with a partner who the participant does not know well and with whom the participant does not intend to share a committed romantic relationship” [12]. The first three items measured participant intentions to engage in casual sex in the next 2 months. An example item read “Over the next 2 months, do you intend to have sex with a casual sex partner?” All items were rated on a 7-point scale with higher scores indicating increased intention to engage in casual sex. A higher total score indicated a greater intention to engage in casual sex. The Cronbach’s α for the three items in this study was 0.89. The next 11 items measured the TPB constructs of casual sex, including participants’ favorable attitudes (five items—for example, “Having casual sex is very unpleasant/very pleasant”; Cronbach’s α = 0.93), subjective norms of acceptance among important persons (three items—for example, “Most people who are important to me think that I should not/I should have casual sex”; Cronbach’s α = 0.69), and perceived control over involvement (three items—for example, “For me, avoiding casual sex is difficult/easy”; Cronbach’s α = 0.82). Each item was rated on a 7-point scale ranging from 1 to 7. For the respective subscales, a higher score indicated a more favorable attitude toward casual sex, higher perceived peer acceptance of casual sex (subjective norms), and higher control over involvement in casual sex.

#### 2.2.2. Parent–Child Discussion on Sexual Issues Scale

We modified the Parent–Child Discussion on Sexual Issues scale developed by Wei [29] (example item: “My father /mother have discussed contraception with me”) into a 24-item scale to measure participants’ experiences of father–child (12 items) and mother–child discussion (12 items). Each item was rated on a 3-point scale ranging from 1 (*never discuss*) to 3 (*frequently discuss*). A higher total score indicated a higher frequency of parent–child discussion. The Cronbach’s α for this scale was 0.93.

#### 2.2.3. Peer Interaction around Sexual Issues Scale

We used the 15-item Peer Interaction Around Sexual Issues Scale developed by Lin [30] to assess three dimensions of peer interaction: influence of peers (example item: “You want to have sex because your peers, classmates, or friends have had sex with the opposite sex”), sharing of romantic experiences (example item: “Your peers share their dating experience with you”), and sharing of sexual experiences (example item: “Your peers share their caressing experience with you”). Each item was scored on a 5-point Likert scale ranging from 1 (*totally disagree*) to 5 (*totally agree*). A higher total score indicated more interactions with peers on sex-related topics. The Cronbach’s α for this scale was 0.93.

#### 2.2.4. Exposure to Sexually Explicit Materials Scale

We modified the Exposure to sexually explicit materials scale developed by Lo et al. [31] into a 28-item scale to measure participants’ frequency of exposure to pornographic media (example item: “You have watched pornographic movies on the Internet in the past year”) and engagement in online sexual activities (example item: “You have had online sexual activity in the past year”). Each item was scored on a 5-point Likert scale ranging from 1 (*never watch/engage*) to 5 (*watch/engage almost every day*). A higher total score indicated a higher frequency of exposure to sexually explicit material and engagement in online sexual activities. The Cronbach’s α for this scale was 0.97.

#### 2.2.5. Demographics

Data on participants’ ages, genders, past sexual experiences, and family structures were collected.

### 2.3. Statistical Analysis

IBM SPSS Statistics 24 (IBM Corp., Armonk, NY, USA) was used to perform the data analyses. The mean scores for intention, the three TPB constructs, parent–child discussion, peer interaction, and exposure to sexually explicit materials were compared between male and female participants and between participants aged 15–19 and 20–24 using the Mann–Whitney *U* test because of a nonnormal distribution in the Shapiro–Wilk test. Because of the presence of multiple comparisons, a two-tailed *p* value < 0.007 (0.05/7) was used to gauge statistical significance. Correlations between variables were examined using Pearson’s correlation. A two-tailed *p* value of <0.002 (0.05/21) was used to gauge statistical significance.

Multiple regression analysis was used to examine the associations of parent–child discussion, peer interaction, and exposure to sexually explicit materials with casual sex intention by controlling for the effects of gender and age. If parent–child discussion, peer interaction, and exposure to sexually explicit materials were significantly associated with casual sex intention, the mediation effects of the three TPB constructs on the associations were further examined using SEM performed with AMOS (v.24, SPSS Inc., Chicago, IL, USA). We adopted Anderson and Gerbing’s two-step strategy to test the hypothesized model [32]. First, confirmatory factor analysis was conducted to assess how well the observed measures reflected the latent constructs. Parent–child discussion, peer interaction, exposure to sexually explicit materials, and TPB constructs related to casual sex demonstrated a good fit. Second, the hypothesized SEM model was tested to examine the relationships among constructs [32]. Multiple fit indexes were used to assess the model fit, including the chi-square (χ^2^) value, degrees of freedom (df), comparative fit index (CFI), and root mean square error of approximation (RMSEA). A satisfactory model fit was indicated by a CFI value greater than or equal to 0.95 and an RMSEA value less than or equal to 0.05 [33]. Some data were found to be nonnormal (skewness was >2), and bootstrapping was used to correct the standard errors [34]. Estimates of path coefficients representing the strength of the paths between two variables were calculated using standardized regression coefficients (i.e., β value).

In order to investigate the indirect effects of parent–child discussion, peer interaction, and exposure to sexually explicit materials on casual sex intention through the mediation of the three TPB constructs, we performed bias-corrected bootstrapping at a 95% confidence interval with 5000 bootstrapping samples and calculated the confidence intervals of the lower and upper bounds; if the lower and upper bounds of the 95% bias-corrected bootstrap confidence interval did not contain zero, the paths between variables were statistically significant [35].

To examine the gender differences, we further tested the invariance of path coefficients across various groups of gender by using multigroup SEM analysis [36]. First, all estimated parameters were constrained to be identical across genders. Subsequently, a non constrained model was examined. Improvement in χ^2^ by freeing the paths among the variables indicated a gender difference. A two-tailed *p* value of <0.05 indicated statistical significance. 

## 3. Results

### 3.1. Demographic Characteristics

Of the 767 participants, 348 were male (45.4%) and 419 were female (54.6%); ages ranged between 15 and 24 years, with a mean age of 17.38 years (standard deviation: 2.26). A total of 140 (18.3%) reported having had sexual experiences prior to the study. Most participants’ fathers (46%) and mothers (58%) had senior high/vocational school education. Approximately 70% of the participants were raised in a nuclear family. Male participants had higher paternal (χ^2^ = 11.8; *p* = 0.003) and maternal (χ^2^ = 7.48; *p* = 0.024) education levels than female participants, whereas no difference in age (*t* = 1.45; *p* = 0.148), past sexual experience (χ^2^ = 0.07; *p* = 0.781), or family structure (χ^2^ = 0.03; *p* = 0.983) was found between male and female participants.

### 3.2. Comparisons of the Variables between Men and Women and between Participants Aged 15–19 and 20–24

The mean scores of the model variables and comparison between men and women and between participants aged 15–19 and 20–24 are listed in Table 1. Significant differences in all the model variables were found between men and women based on the results of the Mann–Whitney *U* test. Men reported a higher intention to engage in casual sex than women did. Men reported more favorable attitudes toward casual sex and a stronger perception of the normative acceptance of casual sex than women did, whereas women reported higher control over involvement in casual sex than men did. Men reported more peer interactions and exposure to sexually explicit materials than women did, whereas women reported more parent–child discussion than men did. Only peer interaction around sexual issues and exposure to sexually explicit materials differed between participants aged 15–19 and 20–24. Those aged 20–24 years reported more peer interaction around sexual issues and exposure to sexually explicit materials than those aged 15–19 did.

### 3.3. Correlation Matrix

Table 2 presents the correlation matrix of the study constructs. The results revealed that favorable attitudes and subjective norms of acceptance were positively correlated with the intention to engage in casual sex, whereas perceived control over involvement was negatively correlated with intention. Three TPB constructs were significantly correlated with each other. Peer interaction and exposure to sexually explicit materials were significantly correlated with intention and the three TPB constructs, whereas the correlations of parent–child discussion with intention and the three TPB constructs were not significant.

### 3.4. Associations of Parent–Child Discussion, Peer Interaction, and Exposure to Sexually Explicit Materials with Casual Sex Intention

Table 3 shows the results of multiple regression analysis examining the associations of parent–child discussion, peer interaction, exposure to sexually explicit materials, gender, and age with casual sex intention. The results support that after controlling for the effects of gender and age, parent–child discussion on sexual issues was negatively associated with the intention to engage in casual sex, whereas peer interaction around sexual issues and exposure to sexually explicit materials were positively associated with the intention to engage in casual sex.

### 3.5. Results of SEM

Given that parent–child discussion, peer interaction, exposure to sexually explicit materials were significantly associated with casual sex intention, the mediation effects of the TPB constructs on the associations were further examined by SEM. The factor loadings (λ) for constructs with multiple indicators ranged from 0.71 to 0.95 in the overall model. The overall model for combined genders fit the data well according to all the fit indices. The overall model resulted in χ^2^ = 253.41, *df* = 210, *p* = 0.83, CFI = 0.98, and RMSEA = 0.02. This model explained 65% of the variance in participants’ intentions to engage in casual sex. As shown in Table 4, the results of the bootstrap test confirmed the existence of a significantly positive direct effect between exposure to sexually explicit materials and casual sex intention. Moreover, there was a negative and significant mediating effect of all three TPB constructs on the association between parent–child sexual communication and casual sex intention. There were positive and significant mediating effects of all three TPB constructs on the associations of peer sexual communication and exposure sexually explicit materials with casual sex intention. The H1 was partially supported and H2 was supported.

**Figure 2:**
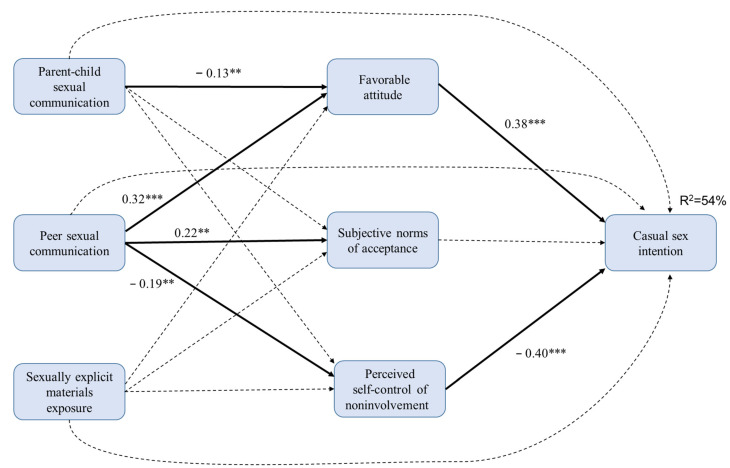
The standard coefficient of men. *** *p* < 0.001; ** *p* < 0.01. Solid lines denote significant paths and dotted lines denote non-significant paths.

We further examined the relationships among parent–child discussion, peer interaction, exposure to sexually explicit materials, three TPB constructs, and casual sex intention in men and women separately. Figure 2 shows the model for men. The model fit was satisfactory: χ^2^ = 254.71, *df* = 210, *p* = 0.83, CFI = 0.99, and RMSEA = 0.03. This model explained 54% of the variance in the casual sex intention of men. Parent–child discussion, peer interaction, and exposure to sexually explicit materials were not directly associated with casual sex intention. Parent–child discussion was negatively associated with casual sex intention via the mediation of decreased favorable attitude. Mother–son sexual discussion was associated with a more negative attitude towards casual sex (*r* = −0.12, *p* = 0.03) among men, whereas father–son sexual discussion had no significant effect. Peer interaction was positively associated with casual sex intention via the mediation of increased favorable attitudes and decreased perceived control over involvement. Exposure to sexually explicit materials was not significantly associated with the intention related TPB constructs.

**Figure 3:**
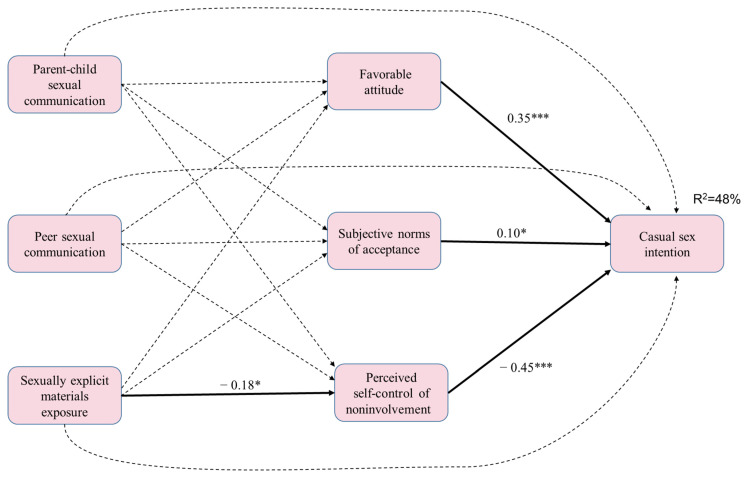
The standard coefficient of women. *** *p* < 0.001; * *p* < 0.05. Solid lines denote significant paths and dotted lines denote non-significant paths.

Figure 3 shows the model for women. The model fit for women was satisfactory: χ^2^ = 250.46, *df* = 210, *p* = 0.83, CFI = 0.99, and RMSEA = 0.02. This model explained 48% of the variance in casual sex intention among women. Parent–child discussion, peer interaction, and exposure to sexually explicit materials were not directly associated with casual sex intention. Exposure to sexually explicit materials was positively associated with casual sex intention via the mediation of decreased perceived control over involvement. Parent–child discussion and peer interaction were not significantly associated with the intention-related TPB constructs.

### 3.6. Gender Differences

The results of the multigroup SEM analysis are presented in Table 5. The results indicated that the associations of peer interaction with subjective norms, perceived control over involvement, and casual sex intention were significantly stronger in men than in women. Moreover, the association of favorable attitudes with casual sex intention was significantly stronger in men than in women. The results supported H3.

## 4. Discussion

The present study found that parent–child discussion, peer interaction, and exposure to sexually explicit materials were significantly associated with the intention to engage in casual sex among adolescents and young adults, though no direct effects were found in the SEM for men and women separately. Moreover, the mediating roles of favorable attitude, perceiving positive social norms toward casual sex, and control over involvement in the associations differed between men and women. For men, decreased favorable attitude mediated the negative association between parent–child discussions and casual sex intention; increased favorable attitudes and decreased control over involvement mediated the positive associations between peer interactions and casual sex intention. For women, decreased control over involvement mediated the positive association between exposure to sexually explicit materials and casual sex intention. Furthermore, the associations of peer interaction with intention to engage in casual sex and its related TPB constructs were stronger in male than in female adolescents and young adults.

### 4.1. Gender Differences in the Intention to Engage in Casual Sex

The present study found that men reported a higher intention to engage in casual sex, more favorable attitudes toward casual sex, and greater perception of the normative acceptance of casual sex than women did, whereas women reported higher control over involvement in casual sex than men did. These gender differences were also found in a previous meta-analysis and were hypothesized to result from gender differences in sexual attitudes, cultural factors, and sexual physiology [37]. A previous study demonstrated that men’s attitudes toward sex were oriented toward game-based love, whereas women’s attitudes toward sex were oriented toward friendship-based love [38]. A sexual double standard exists worldwide; people often praise boys for being sexually active but criticize girls for being simply curious about sex [39]. Furthermore, testosterone may heighten sexual impulses in both men and women; the higher level of testosterone in men may lead to stronger sexual desire than in women [40]. All these factors may contribute to gender differences in the intention to engage in casual sex.

### 4.2. Role of Peer Interaction on Sexual Issues in Intention to Engage in Casual Sex

The present study determined that peer interaction regarding sexual issues was positively associated with the intention to engage in casual sex via the mediations of increased favorable attitudes and decreased control over involvement in men but not in women. Peer group culture may encourage men to relate sexual experiences to peers, display masculinity, and become sexually active [4,22]. However, some scholars argue that the influence of peer interaction on sexual issues depends on the content of the interaction. High-quality peer interactions related to sexual issues such as feeling comfortable, open, and unembarrassed when communicating about sexual issues may have a positive effect on adolescents’ sexual well-being and may increase positive sexual outcomes, such as positive attitudes towards contraception, delayed first sexual encounters, self-efficacy in condom use, sexual satisfaction, and positive emotional responses [41,42]. Layzer et al. argued that peer-based sexual education programs were more effective than traditional programs because peer educators can better understand the situations of young people and allay sexual doubts in an effective manner [43]. It is recommended that health caregivers design peer education activities, such as group discussions over coffee, brainstorming sessions, role-playing, and working through decision trees, to guide thinking about the contextual factors and influences of casual sex.

### 4.3. Role of Parent–Child Discussion in Intention to Engage in Casual Sex

In this study, parent–child discussion was negatively associated with the intention to engage in casual sex via the mediation of decreased favorable attitudes in men but not in women. A sexual double standard persists in Taiwan. Stricter social norms around sexuality exist for women than men; men are generally granted more sexual freedom. Parents of female adolescents mostly emphasize protecting oneself, not having premarital sex, and preventing pregnancy with romantic partners [23]. Our study results also demonstrated that better mother–son sexual discussions indicated a more negative attitude toward casual sex among men, whereas father–son sexual discussions had no significant effect. In the patriarchal society of Taiwan, fathers are treated as the heads of the family, and they mostly play the role of domestic financial supporter. Mothers are largely responsible for communication with their children and caretaking [44]. Future studies should develop interventions to improve parental discussions about sex. In particular, fathers of male adolescents are advised to play the role of a “friend” to offer their sons life lessons and discuss the pros, cons, and effects of casual sex.

### 4.4. Role of Exposure to Sexually Explicit Materials in Intention to Engage in Casual Sex

The SEM for all participants revealed the significantly positive direct effect between exposure to sexually explicit materials and casual sex intention as well as the positive and significant mediating effects of all three TPB constructs on the associations of exposure sexually explicit materials with casual sex intention, although the effect of subjective norm of acceptance was small. In this study, exposure to sexually explicit materials was positively associated with casual sex intention via the mediation of decreased perceived control over involvement among women but not men. Prolonged exposure to pornography among young people leads to a skewed perception of “normal” sexual activity and diminished trust in intimate partners, loss of hope of sexual exclusivity with a given partner, and normalization of promiscuous lifestyles [45]. Women are often objectified as tools for the pleasure of men. Men are usually dominant in pornographic plots, with women often engaging in submissive roles [46]. In one study, frequent consumers of pornography were sexually aroused by, fantasized about, or tried to perform acts seen in pornographic films [47]. Faced with the phenomenon of readily available internet pornography, a top priority for sexual education is to enhance the ability of young girls to critically evaluate pornographic media. Public health professionals concerned about the harm of internet pornography on teenagers developed a pornography literacy program to reduce casual sex [48]. In the program, intervention courses included providing normative ideas about pornography and encouraging critical thinking, self-reflection, role-playing, and re-evaluation of peer beliefs and social norms. Health caregivers should design intervention courses aligned with Taiwan’s cultural characteristics to enhance the ability of girls to think critically about sexual material and notions of physical autonomy and improve their sexual communication skills.

### 4.5. Limitations

This study had several limitations. First, the cross-sectional study design limited the possibility of determining the casual relationships among the variables. Second, this study used a nonprobability sampling method to recruit participants, which might limit the generalizability of the study results. Third, participants might have underreported their intention to engage in casual sex and its TPB constructs due to social desirability. Fourth, this study collected data from adolescents and young adults but not from other sources. The single data source may have resulted in common-method variance. Fifth, parents who did not discuss sex with their children might not agree their children younger than 20 years old to participate in this survey; it might increase the possibility of the bias in sample representation. Sixth, the present study examined the intention to have casual sex; however, some people might have situational casual sex (e.g., in a party, bar, or club where alcohol is involved) and might not have clear intentions to do casual sex. The discrepancy between the intention to have casual sex and real casually sexual behaviors warrants further study.

## 5. Conclusions

Gender differences were identified in the associations between parent–child discussion, peer interaction, and exposure to sexually explicit materials and the intention to engage in casual sex and its related TPB constructs among heterosexual adolescents and young adults; peer sexual communication especially had a stronger positive effect on casual sex intention for men than women. Health professionals should take gender differences into consideration when developing intervention programs to improve sexual health in adolescents and young adults. Furthermore, the TPB constructs of favorable attitudes, subjective norms of acceptance, and perceived control over involvement had various mediating effects on the associations of parent–child discussion, peer interaction, and exposure to sexually explicit materials with the intention to engage in casual sex. These TPB constructs should be considered when designing sexual health programs.

## Figures and Tables

**Table 1:** Comparisons of intention, three TPB constructs, parent–child discussion, peer interaction, and exposure to sexually explicit materials between men and women and between participants aged 15–19 and 20–24: Mann–Whitney *U* test (*n* = 767).

Variable	Range	Overall Mean (SD)	Men (*n* = 348) Mean (SD)	Women (*n* =419) Mean (SD)	*Z*	15–19 years (*n* = 536) Mean (SD)	20–24 years (*n* =231) Mean (SD)	*Z*
Intention to engage in casual sex	3–21	6.56 (4.54)	9.11 (4.79)	4.44 (2.97)	−14.96 ***	6.27 (4.25)	7.23 (5.11)	−1.65
Favorable attitude	5–35	15.23 (8.22)	20.05 (7.31)	11.22 (6.62)	−14.95 ***	14.70 (7.56)	16.45 (9.47)	−1.87
Subjective norm of acceptance	3–21	7.36 (4.40)	8.96 (4.19)	6.02 (4.12)	−9.79 ***	7.35 (4.36)	7.38 (4.51)	−0.21
Self-control of noninvolvement	3–21	16.62 (4.40)	14.74 (4.50)	18.19 (3.64)	−10.76 ***	16.72 (4.15)	16.40 (4.92)	−0.25
Parent–child discussion	24–72	32.96 (8.20)	31.96 (8.60)	33.79 (7.78)	−4.83 **	32.91 (8.40)	33.09 (7.75)	−0.80
Peer interaction	15–75	40.27 (12.57)	44.47 (11.94)	36.78 (12.02)	−8.57 ***	38.48 (12.39)	44.42 (12.03)	−5.78 ***
Exposure to sexual materials	28–140	42.47 (18.65)	52.24 (22.18)	34.36 (9.15)	−14.10 ***	40.85 (17.08)	46.23 (21.45)	−3.57 ***

Note: ** *p* < 0.01; *** *p* < 0.001.

**Table 2:** Correlations between intention, three TPB constructs, parent–child discussion, peer interaction, and exposure to sexually explicit materials: Pearson’s correlation (*n* = 767).

Variables	1	2	3	4	5	6	7
1. Intention to engage in casual sex	-						
2. Favorable attitude	0.65 **	-					
3. Subjective norm of acceptance	0.50 **	0.49 **	-				
4. Perceived self-control of non-involvement	−0.61 **	−0.54 **	−0.47 **	-			
5. Parent–child discussion	−0.11 *	−0.07	−0.09 ^†^	0.09 ^†^	-		
6. Peer interaction	0.29 **	0.33 **	0.22 **	−0.22 **	0.19 **	-	
7. Exposure to sexually explicit materials	0.37 **	0.38 **	0.24 **	−0.32 **	0.12 ^‡^	0.48 **	-

Note: * *p* < 0.002; ** *p* < 0.001; ^†^ *p* < 0.05; ^‡^ *p* < 0.01.

**Table 3:** Associations of parent–child discussion, peer interaction, exposure to sexually explicit materials, gender, and age with casual sex intention: multiple regression analysis (*n* = 767).

Variables	*β*	95% CI
Parent–child discussion	−0.105 **	−0.093, −0.024
Peer interaction	0.115 **	0.016, 0.067
Exposure to sexually explicit materials	0.132 **	0.014, 0.050
Gender (men = 0; women = 1)	−0.401 ***	−4.290, −3.020
Age	0.032	−0.058, 0.188
	*F*= 65.917 ***	
	*R^2^*= 30.2%	

Note. The dependent variable is casual sex intention; *β* is the standardized regression coefficient; CI = confidence interval. ** *p* < 0.01 *** *p* < 0.001.

**Table 4:** Direct, indirect, and total effects of the hypothesized model (*n* = 767).

Paths	Estimate	SE	Bias-Corrected Bootstrapping 5000 Times 95% Confidence Interval	
			Lower	Upper
Direct effectss				
PCC→ Intention	−0.032	0.022	−0.074	0.013
Peer→ Intention	0.018	0.013	−0.008	0.044
Exposure→Intention	**0.022**	0.011	0.001	0.043
Indirect effect				
PCC→Attitude→Intention	**−0.032**	0.013	−0.062	−0.008
PCC→SN→Intention	**−0.005**	0.003	−0.016	−0.001
PCC→PBC→Intention	**−0.037**	0.012	−0.065	−0.016
Peer→Attitude→Intention	**0.041**	0.010	0.023	0.063
Peer →SN→Intention	**0.004**	0.003	0.001	0.012
Peer →PBC→Intention	**0.023**	0.009	0.009	0.046
Exposure→Attitude→Intention	**0.024**	0.007	0.012	0.038
Exposure→ SN→Intention	**0.003**	0.002	0.0001	0.007
Exposure→PBC→Intention	**0.029**	0.006	0.018	0.043
Total effects				
PCC→Intention	**−0.106**	0.032	−0.171	−0.048
Peer→Intention	**0.086**	0.020	0.045	0.127
Exposure→Intention	**0.077**	0.016	0.047	0.109

Note: SN, subjective norm of acceptance; PBC, perceived self-control of non-involvement; PCC, parent–child sexual communication; Peer, peer sexual communication; Exposure, sexually explicit materials exposure. Significant values are in bold. →: indicating the path.

**Table 5:** Comparison of the paths between the models among men and women.

Dependent Variables	Independent Variables	Men		Women		Significance Test of Comparison		
		Estimate	SE	Estimate	SE	ΔDF	Δ*χ^2^*	*p*
Attitude	Parent–child discussion	−0.053	0.025	−0.005	0.031	1	1.367	0.242
	Peer interaction	0.090	0.021	0.036	0.021	1	3.370	0.066
	Exposure	0.008	0.011	0.005	0.023	1	0.014	0.906
Subjective norm	Parent–child discussion	−0.057	0.031	−0.017	0.030	1	0.761	0.383
	Peer interaction	0.069	0.026	−0.002	0.019	1	4.474	0.034
	Exposure	0.001	0.013	0.034	0.025	1	1.258	0.262
Self-control	Parent–child discussion	0.053	0.028	0.029	0.024	1	0.419	0.517
	Peer interaction	−0.059	0.022	0.005	0.015	1	5.448	0.020
	Exposure	−0.022	0.012	−0.047	0.018	1	1.262	0.261
Intention	Attitude	0.482	0.078	0.262	0.041	1	6.280	0.012
	Subjective norm	0.109	0.060	0.076	0.032	1	0.215	0.643
	Self-control	−0.467	0.071	−0.484	0.074	1	0.026	0.872
	Parent–child discussion	−0.032	0.026	−0.011	0.019	1	0.435	0.510
	Peer interaction	0.043	0.022	−0.015	0.013	1	5.209	0.022
	Exposure	−0.002	0.011	0.006	0.014	1	0.202	0.653

## Data Availability

The data generated in this study are available upon reasonable request from the corresponding authors.
